# Research Ethics Boards: Error and Misconception

**DOI:** 10.1371/journal.pmed.0030458

**Published:** 2006-10-31

**Authors:** Angela Bowen

We are compelled to respond to the concerns expressed by Lemmens and Elliott [1] about the Western Institutional Review Board (WIRB) and address the errors and misconceptions contained therein. We have worked diligently to protect the IRB decision-making process from the “for-profit” conflict and many believe that WIRB has set the standard for separation of board and business in the IRB community.

More than 200 people visit WIRB each year to observe our processes, systems, and board meetings. These visitors find that:

- The ethics review process is totally separate from the business of WIRB.
- The regulations are carefully and completely respected on a daily basis.
- Freedom of decision-making is expected by and of each board member.
- Board members and alternates are fully trained and regularly updated.
- Appropriate expertise is available.
- Meetings are convened.
- Disapprovals are as respected as approvals.
- There is never pressure to change a decision.
- WIRB's work comes from the 148 academic and other institutions where WIRB is listed on the Federalwide Assurance form and includes both federally funded and privately funded research from non-institutionally based investigators; about one-third comes from the 400+ public companies, contract research organizations, and foundations that fund medical research.

The following inaccuracies reflect the credibility of the referenced Bloomberg Market article:

- WIRB's annual revenues were not accurately stated.
- The number of Food and Drug Administration (FDA) submission reviews attributed to WIRB was not accurate.
- The Georgia investigators, whom WIRB last reviewed in 1994, were not jailed for endangering research subjects but for diverting funds from the state of Georgia.
- WIRB was not the primary target of the lawsuit cited, which was settled by the insurer as a nuisance settlement on behalf of the group.
- FDA's audits are not done haphazardly; they occur every three years or as indicated.

Auditors, the FDA, the Office for Human Research Protections, and accreditors see our work around the world. Any flaws surely would be noticed and corrected. WIRB will continue to rely on our longstanding reputation for transparency and reliability in the research community.
